# The Editor's Letter-Box

**Published:** 1914-05-02

**Authors:** E. H. Worth

**Affiliations:** Ingleside, 2 Aldrington Road, Streatham, S.W.


*To the Editor of* The Hospital.

Sir,—Dr. Brock misses the whole point of the argument when he says that there are good and bad panel and non-panel doctors. The whole profession signed the pledge, and even before the ink was dry the cowardly section had already began to be affected with nerves, and later, flinging their honour to the wind, accepted terms that they said they would not accept. Surely members of a learned profession ought to have then weighed the consequences before signing a pledge.

It is no use bringing up the plea of economic pressure after putting up such a feeble fight. Why, the old women living in the plains of India would have made a better show!

It is true that the Council of the British Medical Association never gave them a lead when things were looking not well, and neither did the Presidents of the Royal Colleges think it necessary to try and keep them up to the ordinary standard of honour. They let the Lauriston Shaw (*sic*) men do all the advising. I really do not know who I have the most contempt for, the Council of the British Medical Association, the Presidents of the Royal Colleges, or Mr. Lloyd George's medical advisers. Between them they have shaken the faith of the laymen in us to the very foundations.

A City friend of mine lost a bet of £5 on our feeble fight. He said he thought Mr. Lloyd George had at last come up against a body of *men*. He found to his cost that half of us were feeble curs.—Yours truly, E. H. Worth, M.R.C.S.

Ingleside, 2 Aldrington Road, Streatham, S.W. April 25, 1914.

